# Assessment of the *in situ* biomethanation potential of a deep aquifer used for natural gas storage

**DOI:** 10.1093/femsec/fiae066

**Published:** 2024-04-24

**Authors:** Magali Ranchou-Peyruse, Marion Guignard, Pierre Chiquet, Guilhem Caumette, Pierre Cézac, Anthony Ranchou-Peyruse

**Affiliations:** Universite de Pau et des Pays de l'Adour, E2S UPPA, LaTEP, Pau, France; Universite de Pau et des Pays de l'Adour, E2S UPPA, IPREM CNRS UMR5254, Pau, France; Joint Laboratory SEnGA E2S UPPA/Teréga, Pau, France; Universite de Pau et des Pays de l'Adour, E2S UPPA, IPREM CNRS UMR5254, Pau, France; Joint Laboratory SEnGA E2S UPPA/Teréga, Pau, France; Geosciences Department, Teréga, Pau, France; Joint Laboratory SEnGA E2S UPPA/Teréga, Pau, France; Environment Department, Teréga, Pau, France; Universite de Pau et des Pays de l'Adour, E2S UPPA, LaTEP, Pau, France; Joint Laboratory SEnGA E2S UPPA/Teréga, Pau, France; Universite de Pau et des Pays de l'Adour, E2S UPPA, IPREM CNRS UMR5254, Pau, France; Joint Laboratory SEnGA E2S UPPA/Teréga, Pau, France

**Keywords:** deep aquifers, microbial communities, UGS, UHS, underground geological storage, underground hydrogen storage

## Abstract

The dihydrogen (H_2_) sector is undergoing development and will require massive storage solutions. To minimize costs, the conversion of underground geological storage sites, such as deep aquifers, used for natural gas storage into future underground hydrogen storage sites is the favored scenario. However, these sites contain microorganisms capable of consuming H_2_, mainly sulfate reducers and methanogens. Methanogenesis is, therefore expected but its intensity must be evaluated. Here, in a deep aquifer used for underground geological storage, 17 sites were sampled, with low sulfate concentrations ranging from 21.9 to 197.8 µM and a slow renewal of formation water. H_2_-selected communities mainly were composed of the families *Methanobacteriaceae* and *Methanothermobacteriaceae* and the genera *Desulfovibrio, Thermodesulfovibrio*, and *Desulforamulus*. Experiments were done under different conditions, and sulfate reduction, as well as methanogenesis, were demonstrated in the presence of a H_2_ or H_2_/CO_2_ (80/20) gas phase, with or without calcite/site rock. These metabolisms led to an increase in pH up to 10.2 under certain conditions (without CO_2_). The results suggest competition for CO_2_ between lithoautotrophs and carbonate mineral precipitation, which could limit microbial H_2_ consumption.

## Teaser

Effects of H_2_ and CO_2_ on microbial communities in deep aquifers used as geological gas storage: microbiology and tomorrow’s energy mix.

## Introduction

Our societies are facing the challenges of climate change, the need to massively develop renewable energies, the energy sovereignty, and the cost of energy. The ongoing development of the dihydrogen (H_2_) sector and the imminent arrival of green H_2_ from renewable energies in the gas grid (Le Duigou et al. 2017) have led many industrialists and academic researchers around the world to examine the consequences for surface infrastructure (DBI GUT 2017) and underground geological storage (UGS) sites used to balance the grid and secure supplies. Ultimately, the aim is to transform UGS into underground H_2_ storage (UHS; Dopffel et al. 2021, Heinemann et al. 2021, Krevor et al. 2023).

The question of the future of UGS in general, and UHS in particular, is central to (i) developing future massive energy storage to accommodate seasonal variations; (ii) securing countries’ energy reserves; and (iii) avoiding a possible fragmentation of a global gas network that would hinder the development of the H_2_ energy sector or even renewable energies (Rabiee et al. 2021). In the petrochemical and chemical sectors, H_2_ storage in salt caverns has been in use for several decades, and specialists agree that the technology is reliable (Aftab et al. 2022, Réveillère et al. 2022, Bradshaw et al. 2023). However, the number, volume, and geographical distribution of these salt caverns are far from sufficient to store H_2_ on a massive scale, given current production projections, which explains the strong interest in porous reservoirs (Barison et al. 2023). Most of the work focusing on H_2_ storage in porous reservoirs, such as depleted reservoirs or deep aquifers, involves simulations using various models that generally do not take microbial activity into account; several examples of such simulations can be found in the review by Al-Shafi et al. (2023). However, models that take microorganisms into account have shown that microorganisms are likely to have a strong impact on the evolution of these future storage sites (Ivanova et al. 2007, Panfilov 2010, Ebigbo et al. 2013, Amid et al. 2016, Hemme and van Berk 2018, Thaysen et al. 2021, Tremosa et al. 2023).

Many deep environments are home to microbial communities that use H_2_ as an energy source. These communities are referred to by the acronym SLiME, standing for SubLithoautotrophic Microbial Ecosystem (Stevens and McKinley 1995, Fry et al. 1997, Chapelle et al. 2002, Takai et al. 2003, Lin et al. 2005, Crespo-Medina et al. 2014). Several works have demonstrated, directly or indirectly, that UHS in porous reservoirs, particularly in deep aquifers, could lead under certain conditions to *in situ* biomethanation by hydrogenotrophic methanogenic archaea that naturally evolve in these ecosystems (Amigáň et al. 1990, Buzek et al. 1994, Panfilov 2010, Liebscher et al. 2016, Gregory et al. 2019, Strobel et al. 2020, Haddad et al. 2022a, Molíková et al. 2022). These initial biomethanation results obtained for UGS in deep aquifers are attractive because they allow us to envisage a disruptive innovation. UGS in deep aquifers would combine the capture and injection of CO_2_ with the production of nonfossil methane, thereby reducing the consumption of fossil hydrocarbons and curbing the quantities of greenhouse gases released while enabling more virtuous carbon-neutral energy production, i.e. more homogeneous on a territorial scale (Zavarko et al. 2021, Chai et al. 2023). Indeed, deep aquifers are found all over the globe in sedimentary basins, and those in the first 2 km of depth could reach a cumulative volume of 22.6 million km^3^ (Gleeson et al. 2016). The use of these storage sites would be conditional on the presence of a decarbonated H_2_ production area (renewable and nuclear), an accessible source of CO_2_, ideally captured from industry or even the atmosphere (Gutknecht et al. 2018, Hou et al. 2022), and a geological storage reservoir equipped with injection and production wells, as well as a gas network enabling biomethane distribution (Bellini et al. 2022). By overcoming a number of scientific hurdles, these deep, secure sites would represent a biomethanation potential at a scale several times larger than that of conventional catalytic or biological methanation reactors due to the very large reservoir volumes (Molíkova et al. 2022, Vítĕzová et al. 2023).

The concept of biomethanation in porous reservoirs has its origins, on the one hand, in the discovery that part of the methane present in natural gas reservoirs has a biogenic origin (Davis and Updegraff 1954) and, on the other hand, in the realization that *in situ* biomethanation in a geological reservoir could be performed at time-scales compatible with industrial exploitation, as demonstrated by a study of town gas storage in Lobodice (Czech Republic), with an estimated conversion of 17% H_2_ (associated with CO_2_/CO present at the site) to CH_4_ in just 7 months (Šmigáň et al. 1990). In 2021, a study on the Olla Oil Field, which was operated using CO_2_ injection (CO_2_-EOR) until 1986, showed a conversion of 13%–19% of CO_2_ into CH_4_ via methanogenesis (Tyne et al. 2021). At present, three projects are attempting to prove the feasibility of biomethanation in depleted hydrocarbon reservoirs and are being tested under real conditions: the Hychico-BRGM pilot project in Argentina, the Underground Sun Conversion—Flexible Storage Project, and the Bio-UGS Project “Biological conversion of carbon dioxide and hydrogen to methane in porous underground gas storage facilities: Analysis of underground biomethanation potential” (see reviews by Strobel et al. 2020, Dopffel et al. 2021, Bellini et al. 2022).

This study targets a deep aquifer used for natural gas UGS and featuring formation water with low sulfate concentrations (Ranchou-Peyruse et al. 2019). In the context of its potential use as a UHS site, CO_2_ will also be present due to its natural presence in the aquifer, as a coconstituent of the natural gas already present in the storage site or even as a result of voluntary injection (Delshad et al. 2022, Wang et al. 2022). In UHS sites in porous reservoirs, hydrogenotrophic methanogenic archaea compete mainly with sulfate reducers for H_2_ and CO_2_, although a smaller proportion of these substrates may also be consumed by homoacetogens (Haddad et al. 2022b, Mura et al. 2024). Here, cultural and molecular biological approaches were used to assess the effect of the two dominant hydrogenotrophic functional groups in this aquifer, methanogens and sulfate reducers, on gas-phase H_2_. Formation waters from various monitoring wells in the aquifer were collected to (i) assess whether the quantity of methanogens present is a good proxy for the potential for methanogenesis; (ii) evaluate competition and possible inhibition between the two functional groups; and (iii) assess the impact of the rock, specifically calcite, on the metabolic activities of interest.

## Materials and methods

### Sampling campaigns

The deep aquifer used as a UGS site is located in the sedimentary basin of southwest France. The various wells sampled were the subject of a previous study, and the well names have been retained (Ranchou-Peyruse et al. 2019). All formation water sampling was carried out at the head of monitoring wells after they had been purged 10 times with the volume of each well.

**Figure 1.**
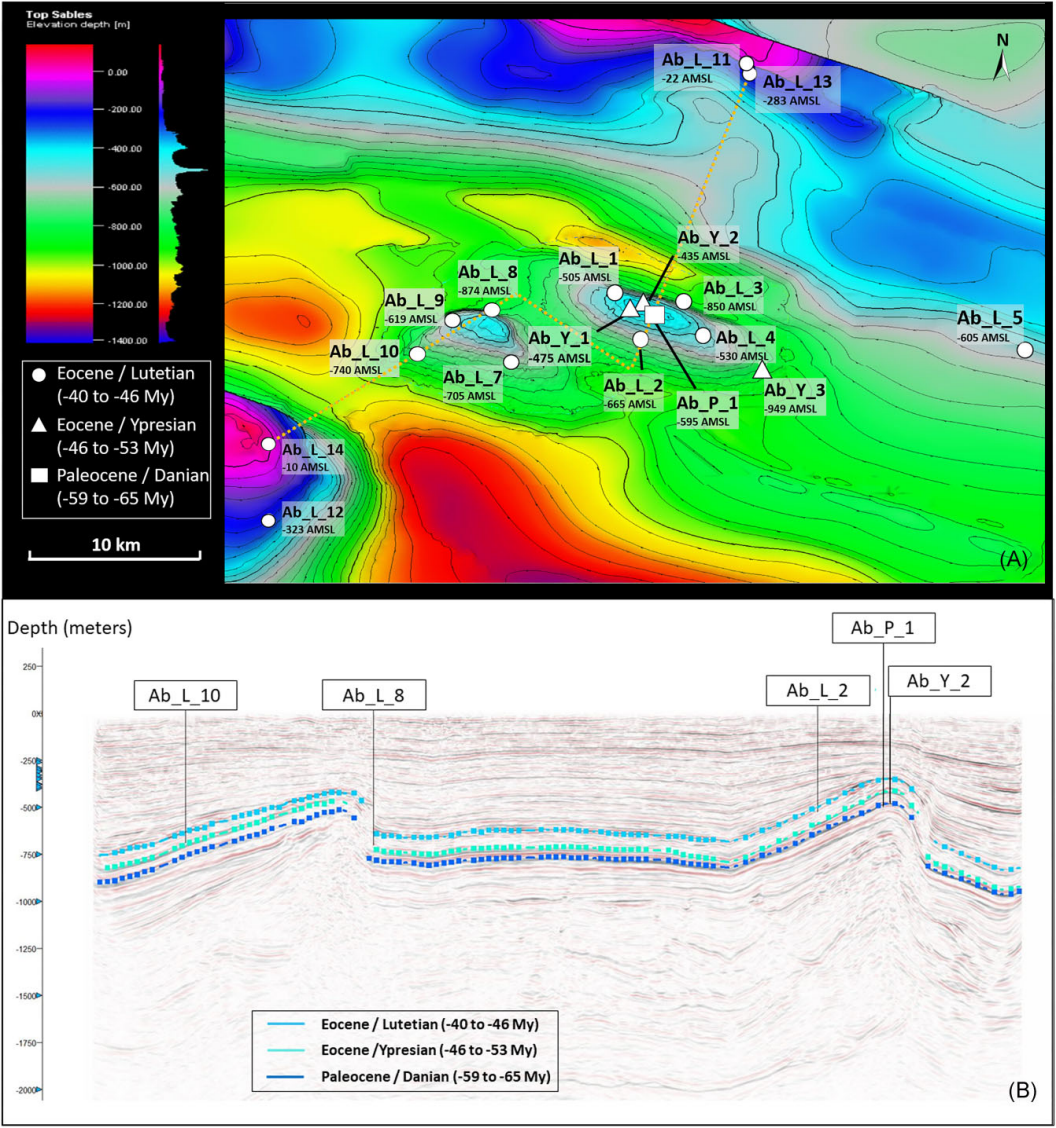
Representations of the deep aquifer targeted for study. (A) Structural map of the aquifer, (B) 2D seismic section of the geological layers constituting the aquifer used as a UGS site along the broken line shown on map A. The interpreted data were acquired from a 3D seismic campaign as well as from older 2D seismic lines acquired in the storage influence zone during oil exploration. For each well, depths are indicated in meters above mean sea level (AMSL).

The first part of the study was carried out using formation water from 17 wells (Fig. 1) sampled between September and October 2020. For each well, the water was collected in four 1 l flasks filled to the brim (three flasks for molecular biology analyses and one for culture methods). Upon return to the laboratory, the water was either stored at 4°C for microbial enrichment (1 l) or filtered in triplicate through 0.1-µm pore size filters (PES, Sartorius Stedim) at a rate of 1 l of water per filter. The filters were then stored at −20°C to preserve the DNA until use.

In the second part of the study, five 1 l bottles of wellhead water were sampled again at seven sites in May and October 2021 based on the results obtained in the first campaign (Ab_L_1, Ab_L_3, Ab_L_7, Ab_L_8, Ab_l_10, Ab_Y_2, and Ab_P_1). For each site, two 1 l flasks were stored at 4°C until used for microbial enrichment. On site, three 1 l flasks were filtered immediately through three different 0.1 µm filters (PES, Sartorius Stedim) and preserved directly in liquid nitrogen until returned to the laboratory, where they were then stored at −80°C until use. The formation water composition and physico-chemical characteristics are presented in Table S1 (Supporting Information) (SOBEGI, Lacq, France).

### Culture methods

Using water sampled during the first campaign, microbial enrichments were performed in 500 ml flasks in an anaerobic chamber (nitrogen atmosphere; Jacomex). A volume of 250 ml of formation water containing the indigenous microorganisms was incubated with a gas phase consisting of H_2_/CO_2_ (80/20; 1.5 bar) at 37°C. For each site, there was only one assay and one abiotic control was carried out by filter sterilizing the formation water (0.1 µm, Sartorius Stedim) inside the anaerobic chamber.

The water sampled at seven sites during the second campaign was subjected to microbial enrichment in a similar way to that described above. This time, several conditions were tested in duplicate for each site: incubation in the presence of a gas phase consisting of either H_2_/CO_2_ or H_2_. Each time, treatments with the presence or absence of calcite (mineral particles <150 µm) were tested. An abiotic control was prepared for each of the conditions tested. For site Ab_P_1, 1 g of crushed cores from the site was used in place of calcite in the presence of H_2_. For site Ab_Y_2, an additional condition was prepared by adding 0.05 g of barite (<150 µm) to the calcite. From the duplicates, one of the tests was dedicated to the analysis of taxonomic diversity, while the second was used to measure the modification of the physico-chemical composition of the water, as well as the effect of incubation on the rock when it was present.

Calcite, reservoir rock, and barite were ground manually using a mortar and pestle before being sieved to <150 µm. The resulting powders were autoclaved before use. Rocks in the abiotic and the biotic replicates reserved for ionic analysis were weighed before and after the experiment using a balance (A&D Company, Limited FZ-5001) with the accuracy of ± 0.001 g.

### Analytical procedures

#### Gas measurement

The evolution of the gas phase (CH_4_, CO_2_, H_2_, and H_2_S) was monitored for all enrichment samples throughout the experiment (GC-µTCD, Micro GC Fusion; Chemlys, France). Measurements were taken in duplicate at each time-point, with an uncertainty of ±5%. Detection limits were 5 ppm for CO_2_ and H_2_ and 20 ppm for CH_4_. The headspace volume was determined for each vial (total vial volume−volume of liquid added). After each gas sample was taken for analysis, the pressure in the flask was measured with a manometer (Digitron 2022P, Farnell). Percent obtained with the GC-µTCD was converted into concentrations (mmol) for each gas. The quantity of gas moles was determined according to the ideal gas law (PV = nRT) with *R* = 8.3145 J mol^−1^K^−1^ and *T* = 310.15 K.

#### pH and Eh measurements

At the initial and final time points, 1 ml was taken from all biotic and abiotic enrichments to measure pH and oxidation–reduction potential (Inlab Ultra-micro ISM and Redox microelectrodes, Mettler Toledo, Seven Compact, Columbus, Ohio, USA).

#### Chemical measurements

For each test condition, water from a biotic replicate and the abiotic control were used in their entirety to quantify ions characteristic of the dissolution or precipitation of minerals of interest (barite, calcite). Ba^2+^ and Ca^2+^ ions were monitored by inductively coupled plasma–atomic emission spectroscopy (ICP–AES) with a limit of quantification of 0.05 mg l^−1^ and 0.1 mg l^−1^, respectively; HCO_3_^−^ and CO_3_^2−^ were determined by titrimetry with a limit of quantification of 40 mg l^−1^ and 60 mg l^−1^, respectively (UT2A, Pau, France). For all enrichment samples, sulfate was measured using ion chromatography (Dionex Integrion HPIC, Thermo Fisher Scientific) with the accuracy of ±5%. To quantify sulfide, 20 µl of cell cultures from each sample were mixed with 480 µl of zinc acetate at 2% concentration for sulfide quantification (Cline 1969). A standard curve was constructed using a sulfide solution, and along with the samples, 200 µl of DMPD (*N, N*-dimethylparaphenylenediamine sulfate) at 0.2% (w/v) were added After 20 min in the dark at room temperature, the absorption at 670 nm was measured with a V-1200 spectrophotometer (VWR).

#### Molecular approaches

During the first campaign, molecular analyses were carried out only on DNA extracted from formation water filtered in the laboratory (0.1 µm, Sartorius, Stedim). The *16S rRNA* (v4–v5), *dsrB* and *mcrA* genes were quantified to estimate the presence of all prokaryotes, sulfate reducers and methanogenic archaea, respectively. During the second campaign, the water was filtered directly on site (0.1 µm, Sartorius, Stedim), and the filters were preserved in liquid nitrogen until they reached the laboratory, where they were stored at −80°C until use.

#### Nucleic acid extraction and RT-PCR

All filters were ground manually using a mortar and pestle with liquid nitrogen. For samples from the first sampling campaign, DNAs were extracted using the DNeasy Power Soil kit (Qiagen) according to the supplier’s recommendations. For the second part of the study, all nucleic acids were extracted using the Fast RNA Prosoil Direct kit (MP BIO Medicals), and DNAs and RNAs were separated using the All Prep RNA/DNA kit (Qiagen), according to the supplier’s recommendations. Nucleic acids were quantified using Quant-it^TM^ dsDNA HS (High sensibility) and Quant-it^TM^ RiboGreen kits (Invitrogen). RNAs were converted to cDNAs using the M-MLV reverse transcription kit (Invitrogen) according to the supplier’s recommendations.

#### PCR, qPCR, and sequencing

From the DNAs and cDNAs obtained, the *16S rRNA, dsrB* and *mcrA* genes were targeted using the primer pairs 515F-928R, dsr2060F-dsr4R, and mlasF-mcrAR (Wagner et al. 1998, Geets et al. 2006, Steinberg and Regan 2008, 2009, Wang and Qian 2009). To reduce inhibition, bovine serum albumin (BSA, NEV-B9200S) was used in the PCRs at a concentration of 1 mg ml^−1^. For amplification of the *16S rRNA* and *dsrB* genes, the Taq PCR kit (Roche) was used, while for the *mcrA* gene, the Fidelio® Hot Start PCR kit (Ozyme) was used. The procedures have previously been described in more detail in Haddad et al. (2022b).

Genes, their transcripts and associated standards were quantified by quantitative PCR (qPCR; Biorad CFX Connect) with 41 Takyon NO ROX SYBR 2X MasterMix blue dTTP (Eurogentec), as previously described (Haddad et al. 2022b).

High-throughput sequencing was performed using MiSeq 2 × 250 bp technology according to the manufacturer’s instructions by the GenoToul genomics platform in Toulouse, France. Each primer pair contained the adapters GTGYCAGCMGCCGCGGTA (forward) and CCCCGYCAATTCMTTTRAGT (reverse). The raw sequencing data are publicly accessible on NCBI SRA under Bioproject ID PRJNA1051807. For global diversity analysis, the MiSeq sequencing results were processed using QIIME 2 (Bolyen et al. 2019, version2022.11). Amplicon sequence variants (ASVs) were generated by DADA2 (Callahan et al. 2016) after sequences had been demultiplexed, filtered, denoised, trimmed of any nonchemical sequences, and filtered for singletons. Taxonomic affiliation was performed on the SILVA v138 database (Quast et al. 2012, Yilmaz et al. 2014). The *mcrA* and *dsrB* sequences were processed as for the 16S rRNA sequences. Affiliation of *mcrA* ASVs was based on the database of Yang et al. (2014). For *dsrB* sequences, ASVs were affiliated with our own database. The *dsrB* database comprises 1089 sequences, including 46 Euryarchaeota, 9 Actinobacteria, 20 Chlorobi, 259 Firmicutes, 6 Nitrospirae, 389 Proteobacteria, 1 Spirochaetas, 1 Synergistetes, 15 Thermodesulfobacteria, 1 Verrucomicrobia, and 342 Reductive_Bacteria_type_DsrAB from the database of Pelikan et al. (2016). Calculations and analyses were performed in R.Studio (version 4.2.2) using the Phyloseq (McMrudie and Holmes 2013) and ggplot 2 (Wickham 2016; https://ggplot2.tidyverse.org/) packages. For heatmaps, the ComplexHeatmap (Gu 2016, 2022) package was used; for PCA and PCoA, the Corrplot (Wei and al. 2017; https://github.com/taiyun/corrplot), FactorMineR (Lê et al. 2008) and factoExtra (Kassambara and Mundt 2020; https://cran.r-project.org/web/packages/factoextra) packages were used. Distance calculations were performed with Bray‒Curtis for PCoA. For PCA, we used the analysis of covariances.

## Results and discussion

### Targeting UGS sites that are already functional for upgrading to UHS sites

There is an urgent need to assess the potential of the various aquifers used as UGS sites to determine their possible future uses (H_2_ storage, CO_2_ storage, *in situ* biomethanation, geothermal energy, and so on). For this study, we selected an aquifer used for natural gas storage (Fig. 1) that has already been the subject of several studies and could be converted into a UHS site (Ranchou-Peyruse et al. 2019, Haddad et al. 2022a). The aquifer is configured into two anticlines (Fig. 1A, in turquoise blue in the center of the image; Fig. 1B), which enables two storage zones to be accommodated in the submolassic sand layer composed mainly of quartz, some calcite, and occasionally dolomite and K-feldspar (André et al. 2002), with a porosity varying between 25% and 35% and a low concentration of sulfate (from 21.9 to 197.8 µM; Table S1, Supporting Information); consequently, the site is *a priori* favorable for H_2_ storage (Bo et al. 2021). Of the 17 sampling sites, 13 are located in the Eocene-Lutetian stratum dated from −40 to −46 My and coded Ab_L_1 to Ab_L_14 (except Ab_L_6) at depths ranging from −10 to −874 m above mean sea level (AMSL). Three wells provide access to water from the lower Eocene-Ypresian level (−46 to −53 My) at depths ranging from −475 to −949 m AMSL. Finally, formation water was sampled in the lower Paleocene/Danian layer (−59 to −65 My) at −595 m AMSL; stored gas does not reach this layer, and a new UHS site could be considered here. The average age of the water circulating in this zone has been estimated by the GAIA project using ^14^C and some ^36^Cl dating to be between 20 000 and 50 000 years old (http://infoterre.brgm.fr/rapports/RP-69126-FR.pdf), with pore-level circulation estimated at ∼5 m year^−1^ (Labat 1998).

### Quantification of sulfate-reducing and methanogenic microorganisms at all sites to assess biomethanation potential

**Figure 2.**
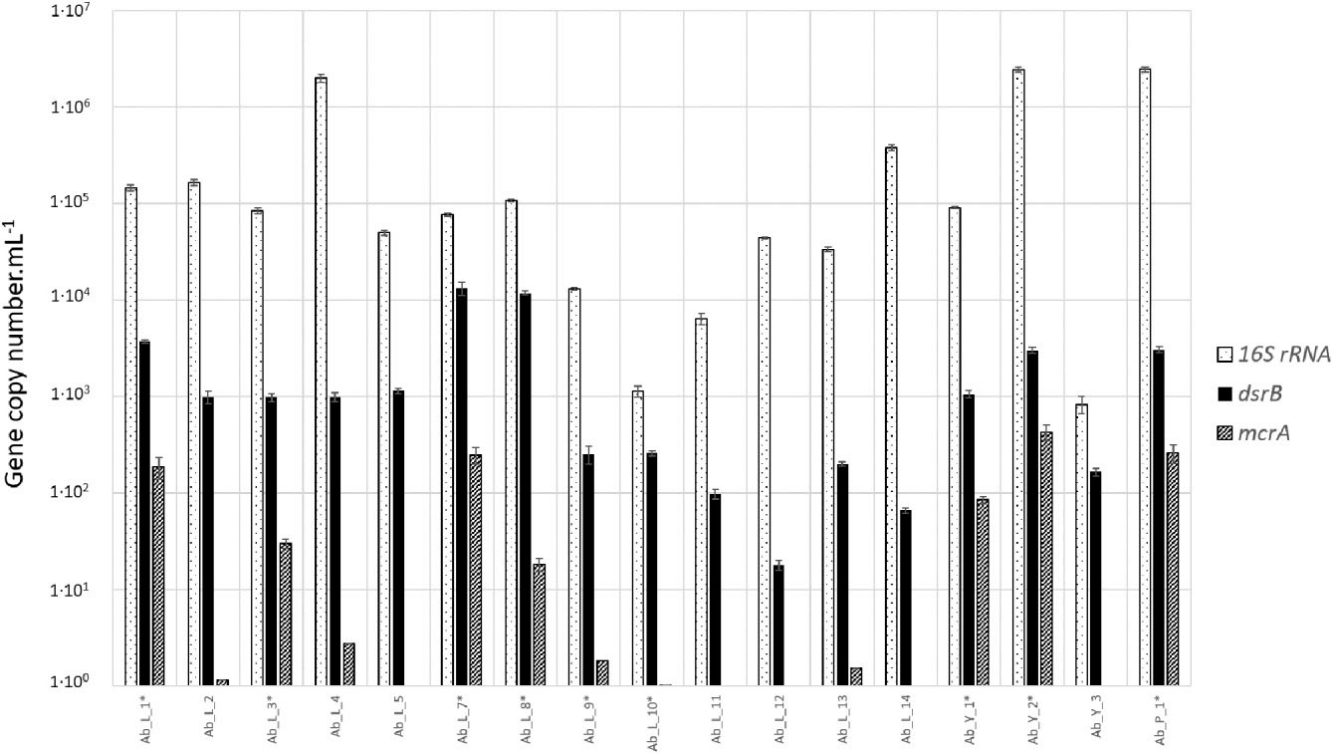
Comparison of prokaryote (bacteria and archaea) quantifications in the 17 formation waters sampled from the three levels of the aquifer. Concentrations of prokaryotes, sulfate reducers, and methanogens were estimated by qPCR in copy numbers per milliliter of water of the *16S rRNA, dsrB*, and *mcrA* genes, respectively. *: formation water that showed methane production after incubation in the presence of H_2_/CO_2_ (80/20; 1 bar).

An initial sampling campaign was carried out in October 2020 to screen the 17 sites for their estimated *16S rRNA* gene copy concentrations (total prokaryotes), *dsrB* (sulfate reducers) and *mcrA* (methanogens), as presented in Fig. 2. For prokaryotes, the average concentration for all sites studied was 4.8 × 10^5^ ± 3.3 × 10^4^ copies of the *16S rRNA* gene ml^−1^, with the lowest concentrations found at sites Ab_L_10 and Ab_Y_3 with 1.1 × 10^3^ ± 1.5102 and 8.3 × 10^2^ ± 1.7 × 10^2^ copies of the *16S rRNA* gene ml^−1^, respectively. Conversely, the sites with the highest concentrations were Ab_L_4, Ab_Y_2, and Ab_P_1, with 2.1 × 10^6^ ± 1.9 × 10^5^, 2.4 × 10^6^ ± 1.5 × 10^5^, and 2.5 × 10^6^ ± 1.5 × 10^5^ copies of the *16S rRNA* gene ml^−1^, respectively. All sites showed the presence of sulfate-reducing microorganisms, which often dominate microbial communities. On the other hand, based on *mcrA* gene detection and quantification, the presence of methanogens was observed at only 12 sites with variable and low concentrations ranging from 1.6 × 10^0^ to 4.3 × 10^2^ ± 8.3 × 10^1^  *mcrA* gene copies ml^−1^. The corresponding formation waters were also incubated in the laboratory with a mixture of H_2_/CO_2_ (80/20; 1 bar), and the asterisk in Fig. 2 identifies the samples showing methanogenesis activity: Ab_L_1 (2% of CH_4_ in 49 days of incubation), Ab_L_3 (6% in 40 days), Ab_L_7 (6.2% in 32 days), Ab_L_8 (0.7% in 17 days), Ab_L_9 (0.2% in 254 days), Ab_L_10 (2.8% in 193 days), Ab_Y_1 (2.7% in 48 days), Ab_Y_2 (1.9% in 138 days), and Ab_P_1 (3.9% in 97 days). As expected, the absence of detection of the *mcrA* gene in water was corroborated by a systematic absence of methanogenesis, and quantification of this gene is therefore a good proxy for this metabolic capacity. Of the 12 formation water samples with the *mcrA* gene, nine showed methane production. Over a 1-year monitoring period, samples Ab_L_2, Ab_L_4 and Ab_L_13 showed no methane production. Low concentrations of *mcrA* gene copies alone cannot explain these latest results, since methane production in other assays was sometimes achieved at lower concentrations. We therefore hypothesize that the methanogens encountered at these three sites, such as members of the families *Methanomicrobiaceae* and *Methanosarcinaceae*, could be nonhydrogenotrophic and use acetate, formate, alcohols and methylated compounds identified at some sites in this aquifer (Ranchou-Peyruse et al. 2019). This could also imply a low acetogenic activity (i.e. production of acetate and/or formate), preventing sustained activity of acetotrophic methanogens.

It is important to note that, with the exception of sites Ab_L_2 and Ab_L_4, all the other sites that did not exhibit methanogenic archaea and/or methane production are remote from current gas storage locations (Fig. 1A). The redox potential may partly explain some of these results, since several of these sites had redox potential unfavorable to hydrogenotrophic methanogenesis, such as Ab_L_5 (−73.9 mV), Ab_L_11 (−71.2 mV), Ab_L_12 (+130.9 mV), and Ab_L_13 (−50.1 mV), instead of the optimal −200 to −400 mV (Reeburgh 1983, Hirano et al. 2013). Ab_L_12 showed great variation in redox potential over the years, ranging from −119.0 to 161 mV since June 2020.

Based on the results of this first sampling campaign, a panel of sites was selected for further study: Ab_L_1, Ab_L_3, Ab_L_7, Ab_L_8, Ab_Y_2, and Ab_P_1. The choice took into account the geological layer of the formation water (L: Eocene-Lutetian, Y: Eocene-Ypresian, and P: Paleocene-Danian), methane production from the H_2_/CO_2_ gas mixture, and the quantity of *mcrA* genes. On this last point, site Ab_L_10 was also selected as it had few copies of *mcrA* genes but nevertheless showed methanogenesis potential in cultivation trials. The rest of the study focused on demonstrating the effect of physico-chemical and microbiological parameters on methanogenesis.

### Physico-chemistry of water samples from seven selected sites

Two new sampling campaigns were carried out in May and September 2021 to resample the seven selected formation waters (Table S1, Supporting Information). These waters had low salinity characterized by an electrical conductivity of ∼300 µS cm^−1^ and negative redox potential between −40.3 and −351 mV. Average sulfate concentrations ranged from 21.9 to 197.8 µM. Nitrate and nitrite concentrations were all below the detection limits of 1.6 µM and 0.3 µM, respectively. For dissolved iron, we were unable to distinguish between Fe^2+^ and Fe^3+^, but the redox potential at the sites favors its more reduced form. André et al. (2007) suggested an equilibrium between Ca-HCO_3_ facies and the dissolution of carbonate minerals such as calcite (CaCO_3_). These carbonates may represent a source of carbon accessible to autotrophic microorganisms under the pH and redox potential conditions prevailing in the aquifer. There are complex balances between carbonate minerals, CO_3_^2−^ and HCO_3_^−^, CO_2_ dissolved in water and CO_2_ in the gas phase (gas storage). By consuming dissolved CO_2_, lithoautotrophic microorganisms significantly alter these balances. The metabolic groups likely to dominate microbial communities are sulfate reducers, methanogens, homoacetogens and fermenters. Given the nature of our study (i.e. *in situ* biomethanation), we decided to focus on sulfate reducers and methanogens. These two functional groups comprise H_2_-consuming lithoautotrophic organisms. We consider homoacetogens and fermenters as complementary, but nonetheless secondary, in the functioning of these communities in the context of massive H_2_ injection. Indeed, it is expected that homoacetogens will consume part of the H_2_ and CO_2_ to form acetate and/or formate (Stoll et al. 2018, Haddad et al. 2022b, Mura et al. 2024), which will be consumed by the rest of the microbial community and, in particular, by methanogenic archaea to form methane (Pan et al. 2016) and heterotrophic sulfate reducers (Weijma et al. 2002, Dai et al. 2022). The experiment carried out by Haddad et al. (2022b), which aimed to simulate H_2_ injection into an aquifer similar to the one in this study in terms of sulfate concentration (around 150 µM), showed that the main consumers of H_2_ were methanogens (around 80% of the H_2_), while homoacetogens accounted for only 4%. The remaining H_2_ lost (around 16%) could be considered as consumed by sulfate reducers. In these oligotrophic environments, where organic carbon concentrations are low (1.1 mg l^−1^) or below the detection limit, the impact of fermenters is expected to be strongly constrained, participating in particular in the recycling of microbial necromass into H_2_, CO_2_, and other organic acids that can be used by sulfate reducers and methanogens. Finally, the low detected concentrations of ammonium (between 3.3 and 26.6 µM) and dissolved phosphates (<2.1 µM) suggest a low capacity of these ecosystems to sustain a much higher biomass concentration than before H_2_ injection. These molecules have already been cited several times as limiting nutrients in the deep biosphere (Madigan et al. 1997, Head et al. 2003). Similarly, the concentrations of certain metals such as nickel, essential for the proper functioning of enzymes such as hydrogenases, could be limiting factors for hydrogenotrophic microbial populations in these environments. This information is crucial and should be taken into account in future UHS modeling incorporating the microbiological dimension (Hagemann et al. 2016). In our case, we believe that with a slow recharge of the aquifer (5 m year^−1^; Labat 1998) and in the context of a rock overwhelmingly composed of quartz and few minerals that could serve as a source of phosphorus or nitrogen, the cell concentration will remain constant to within one log. Unsurprisingly, these two parameters (i.e. ammonium and phosphate) do not appear to be the only ones driving the communities, since their concentrations were not directly correlated with those of prokaryotes in the collected formation water (Table S1, Supporting Information; Fig. 2).

### Enrichment in the presence of H_2_/CO_2_ gas phase (80/20; 1 bar)

Following this first part of the study, incubations in the presence of calcite (CaCO_3_) were carried out. Calcite plays a role in methanogenesis as an indirect carbon source via its dissolution, but its presence in the geological structures used for storage can lead to changes in porosity and permeability (Haddad et al. 2022b, Saeed et al. 2023). In these UGS sites, CO_2_ is present naturally or artificially (coinjected with natural gas to ∼2%; Burgers et al. 2011). Added to this is a complex balance between gaseous and dissolved CO_2_, on the one hand, and between carbonates (CO_3_^2−^) and bicarbonates (HCO_3_^−^) in water and carbonate minerals on the other. Cultivation trials were carried out at near-atmospheric pressure (1 bar at the start of the experiment) in flasks to screen a wide range of conditions, which would not be possible with pressurized experiments. The pressures encountered on these sampling sites (between 40 and 80 bars) are relatively low, compared to abyssal pressures, e.g. and are thought to have little effect on the microorganisms that evolve there; no piezophile has ever been discovered in deep continental systems. *A priori*, the microorganisms revealed in this study at atmospheric pressure would be the same at pressures simulating those *in situ* (i.e. high pressure). On the other hand, it is certain that manipulations at high pressure have an effect on the solubility of gases in water and therefore their accessibility to microbial populations, particularly in the case of lithoautotrophs. Here, the aim was not to assess yields but rather to evaluate a potential for hydrogenotrophy. For each site, the formation water and its indigenous microbial community (without nutrient supplementation) were brought into contact with a gas phase of H_2_/CO_2_ (80/20) or H_2_ alone, with or without calcite. In the case of Ab_Y_2, an additional condition was added with the presence of barite (BaSO_4_) as a potential sulfate source for sulfate reducers (Haddad et al. 2022b). For Ab_P_1, calcite was replaced with rock from the reservoir to mimic *in situ* conditions as closely as possible.

**Figure 3.**
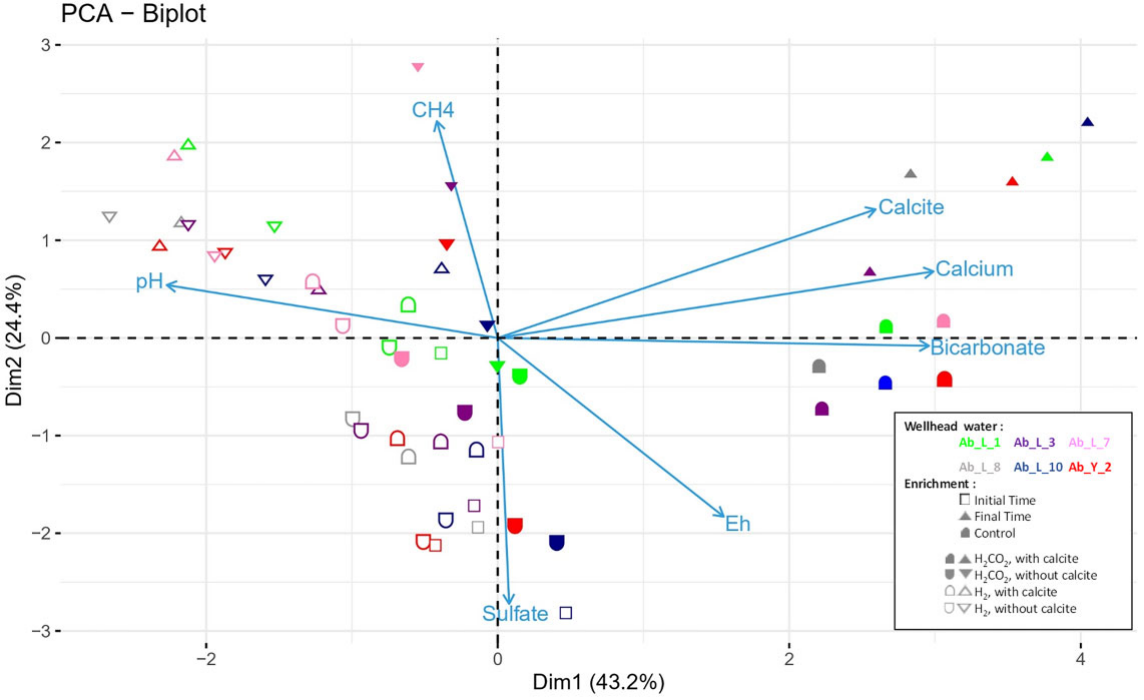
PCA of the main physico-chemical parameters (bicarbonate, calcium, calcite, CH_4_, pH, *Eh*, and sulfate) before and after incubation of the microbial communities from the six formation waters studied (Ab_L_1, Ab_L_3, Ab_L_7, Ab_L_8, Ab_L_10, and Ab_Y_2). Incubations were carried out in the presence of a gas phase consisting of either H_2_/CO_2_ (80/20; 1 bar) or H_2_ only and with or without calcite. The results from site Ab_P_1 are not shown in this figure (Fig. S4, Supporting Information).

The most critical and quantifiable physico-chemical data measured at the start of the experiment and after 26 to 193 days of incubation are represented in the principal component analysis (PCA) shown in Fig. 3, explaining 67.6% of the sample distribution. As expected, the “Bicarbonate,” “Calcium,” and “Calcite” vectors are associated and correlate very well with Axis 1. They aggregate the controls and the H_2_/CO_2_ tests (both with calcite) at the end of the experiment. The “sulfate” vector correlates well with axis 2 and is opposite to the “CH_4_” vector. The almost right angle formed by the “sulfate” vector and the group of the three “bicarbonate”–“calcium”–“calcite” vectors indicates that these two sets of variables are independent of each other. Finally, the “*Eh*” vector is logically opposed to the “CH_4_” vector. A summary of the information from the seven analyzed variables shows that, regardless of the conditions tested, sulfate is the factor with the greatest influence at the start of incubation (as indicated by the empty geometric shapes in Fig. 3). While the sulfate concentration remained constant in all the abiotic controls, sulfate reducers consumed sulfate in all the biotic trials, although this consumption was not total over the incubation period (Table S2, Supporting Information). All biotic tests showed methane production and the presence of sulfate at the end of the experiment, suggesting that methanogens were able to thrive and be active at the same time as sulfate reducers (Table S3, Supporting Information). Black iron sulfide precipitates were observed in all biotic assays. In the conditions without rock and without barite, the sulfide concentrations did not exceed 5 µM at the end of incubation. With the rock from the site (Ab_P_1), these concentrations increased from 2.3 ± 1.3 µmol of sulfide to 11.3 ± 0.9 µmol of sulfide. For the Ab_Y_2 formation water, the sulfide production increased from 2.9 ± 1.6 µmol of sulfide to 7.2 ± 0.0 µmol with the barite supplementation (Ab_Y_2) without there being any more sulfate-reducing agents quantified (Table S3, Supporting Information). Low sulfate concentrations allow methanogens to compete for H_2_ with sulfate reducers. In their work, Lupton and Zeikus (1984) set a concentration limit of ∼5 mM, well above the concentrations found at the various sites in the present aquifer. H_2_ tests, with and without calcite, are grouped together in the upper left quadrant (Fig. 3). These trials are strongly marked by their highest pH values, since the acidity generated by CO_2_ solubilization from the gas phase is absent, and calcite dissolution was observed. Clearly, in the condition with CO_2_ in the gas phase, CO_2_ was the carbon source for methanogens and other chemolithoautotrophs. In the condition without CO_2_ but with calcite, calcite dissolution enabled methanogenesis (CaCO_3_ ↔ CO_3_^2−^/ HCO_3_^−^ ↔ CO_2(aq)_). Biotic tests with H_2_ alone in the gas phase naturally showed higher initial pH values (average pH 8.1 ± 0.1) than those under H_2_/CO_2_ conditions (average pH 6.3 ± 0.2). At the end of incubation in CO_2_-free conditions with calcite, the highest pH was 10.2, averaging 9.3 ± 0.7 across all sites. The ionic Ca^2+^ concentration decreased under the action of microorganisms (0.93 ± 0.08 mM in the abiotic controls versus 0.32 ± 0.19 mM under biotic conditions). Initially, calcite-carbonate-dissolved CO_2_-gas equilibrium was achieved. The methane production indicates that the dissolved CO_2_ was consumed by methanogenic archaea. This consumption led to increased calcite dissolution, the release of calcium ions and an increase in pH with the appearance of hydroxyl ions. Methanogenesis and sulfate reduction are associated with alkalinization (Berta et al. 2018, Dopffel et al. 2023), and this can lead to conditions deviating from the optimal growth conditions of methanogenic archaea, which are generally at approximately pH 6.5 to 8.5, but their resistance can reach pH 10 for some (Gerardi 2003, Liu and Whitman 2008, Thayssen et al. 2021). In the assays without CO_2_, we assume that when the pH of the enrichments became very alkaline, conditions became unfavorable for microorganisms, but not necessarily because of a toxic effect on microorganisms, as has already been observed (Bassani et al. 2015). Calcium ions could then complex with the organic matter of the necromass (Kloster et al. 2013, Zhang et al. 2019), contributing to lower concentrations of this ion in biotic tests than in abiotic tests (Table S2, Supporting Information). This hypothesis will require dedicated experiments to confirm or disprove it under experimental conditions simulating environmental parameters, in particular those related to pressure, salinity, temperature, rock type, and microorganisms. In the context of *in situ* biomethanation, this point is crucial, as it assumes that during methanogenesis, alkalinization initiates a new thermodynamic equilibrium that induces competition for CO_2_ between lithoautotrophs (i.e. methanogens, sulfate reducers, and homoacetogens) and carbonate precipitation. After the depletion in sulfate, this would represent a potential brake on methanogenesis and imply a possible decrease in porosity/permeability as a function of Ca^2+^, Mg^2+^, or Fe^2+^ concentration, which would induce calcite (CaCO_3_), magnesite (MgCO_3_), or siderite (FeCO_3_) precipitation, respectively. For the CO_2_- and calcite-free conditions, we can only hypothesize CO_2_ production by fermentative and heterotrophic functional groups (i.e. sulfate reducers) growing on the necromass of part of the microbial community.

### Methane production as a function of test conditions

**Figure 4.**
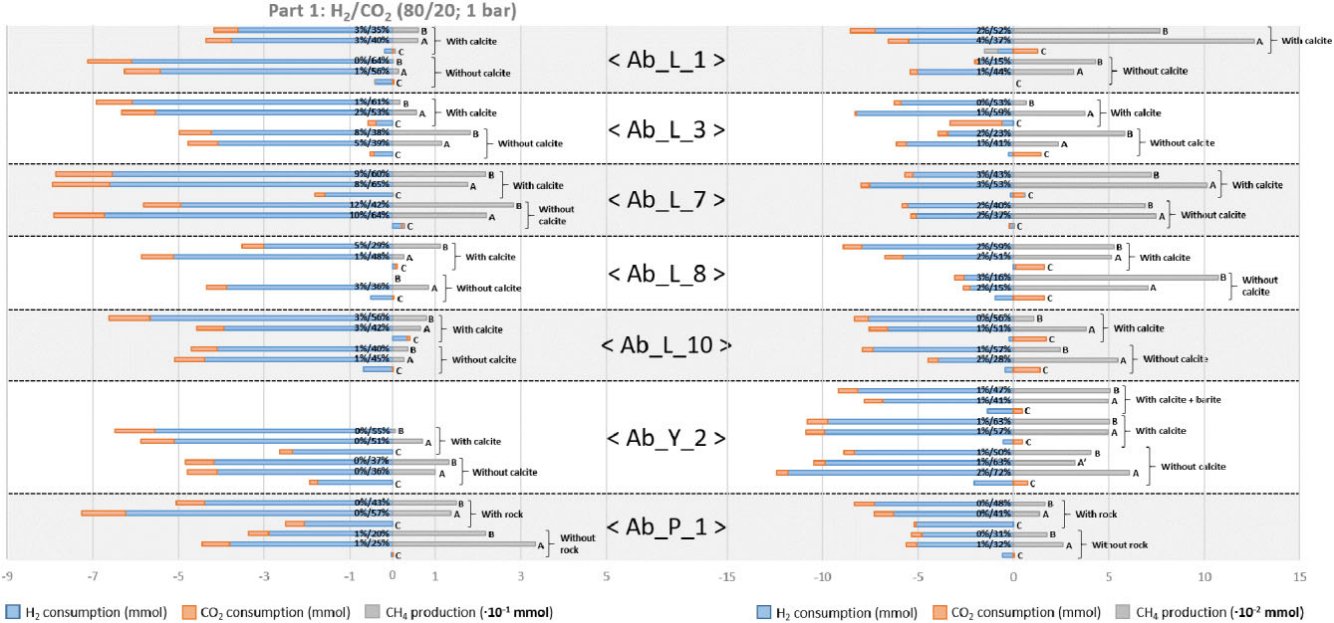
Monitoring of the gas phase evolution in microbial tests on the seven formation waters with and without calcite. Part 1: tests in the presence of a H_2_/CO_2_ gas phase (80/20; 1 bar); Part 2: tests in the presence of a H_2_ gas phase (1 bar). Test Ab_Y_2 also featured an additional condition with added barite (BaSO_4_). Test Ab_P_1 was carried out with aquifer rock rather than calcite. C: abiotic controls; A/A’: trials used for molecular biology analysis; B: trials used for physicochemical analysis. X%/Y%: written on histograms; X% indicates the theoretical percentage of H_2_ consumed by methanogens as a function of the number of mmol of CH_4_ produced based on the hydrogenotrophic methanogenesis reaction (4H_2_ + CO_2_ → CH_4_ + 2H_2_O); Y% indicates the theoretical percentage of H_2_ consumed by other hydrogenotrophic microorganisms (total H_2_ disappeared—H_2_ consumed by methanogens).

**Figure 5.**
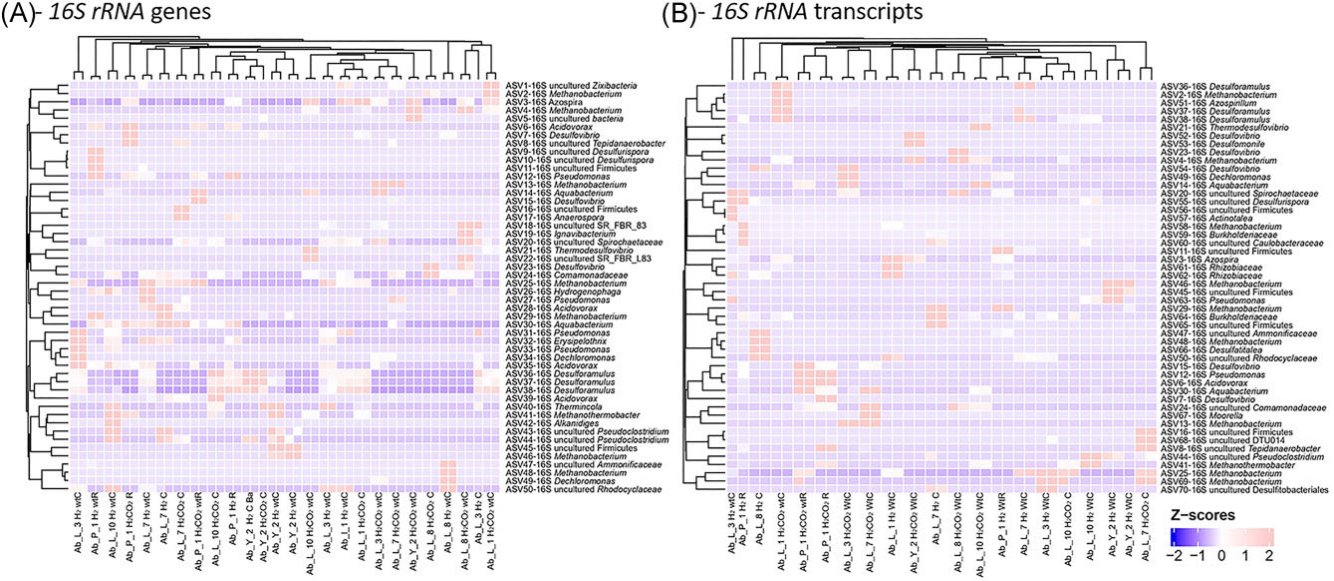
Taxonomic diversity of prokaryotes based on the *16S rRNA* gene in different enrichment cultures at the end of incubation in the presence of H_2_. (A) Heatmap representing taxonomic diversity results based on the *16S rRNA* gene. The 50 dominant phylotypes, representing between 86% and 99% of sequences in each culture trial, are indicated. (B) Heatmap showing the taxonomic diversity results based on *16S rRNA* gene transcripts. The 50 dominant phylotypes, representing between 83% and 99% of sequences in each cultivation trial, are shown. H_2_: incubation with H_2_ in the gas phase; H_2_CO_2_: incubation with H_2_/CO_2_ (80/20; 1 bar) in the gas phase; C: incubation with calcite; Wt: incubation without calcite; R: incubation with rock; WtR: incubation without rock; and Ba: incubation with barite.

All the test conditions for the seven formation waters, apart from the abiotic controls, showed methane production (Fig. 4). It should be noted that in the formation waters closest to the stored natural gas bubble (Ab_L_1 and Ab_L_7), methane may still have been dissolved when the experimental tests began, which explains some of the results. The highest methane production was observed under H_2_/CO_2_ conditions (80/20) and without calcite (CaCO_3_; Fig. 4, Part 1). Methane production was also observed for Ab_L_10 formation water, which had a barely detectable amount of *mcrA* genes. Logically, in incubations with only H_2_ in the gas phase (Fig. 4 Part 2), methanogenesis was generally less efficient than in the presence of H_2_/CO_2_ (80/20). In all these assays, an increase in pH was measured, from around 8.0 at the start of the incubations to 10.2 (in particular, Ab_L_7 with calcite). The methanogenesis in the tests without calcite (and without CO_2_) implies that a significant proportion of the carbon used to produce methane did not come from calcite. We hypothesize that the source carbon could be bicarbonate ions in the waters, with concentrations ranging from 2.5 to 3.2 mM (Table S2, Supporting Information), and by the fermentation and heterotrophy of the microbial necromass, producing H_2_, CO_2_, and organic acids that feed methanogenic archaea. Ab_Y_2 formation water in the presence of barite (BaSO_4_), a potential source of sulfate, did not show an increase in the concentration of the sulfate reducers (Table S3, Supporting Information), but rather in their activity (i.e. more sulfide produced). We deduce that for such an aquifer with relatively low sulfate concentrations between 0.02 and 0.2 mM (Table S2, Supporting Information), methanogenesis can take place at the same time as sulfate reduction, and the latter is not limiting for the development of methanogens. Based on the hydrogenotrophic methanogenesis reaction (4H_2_ + CO_2_ → CH_4_ + 2H_2_O) and the quantities of methane detected at the end of incubation, the theoretical H_2_ consumption by this metabolism has been estimated at between 0.1% and 13.4% of the H_2_ consumed under H_2_/CO_2_ conditions, and between 0.3% and 3.8% under conditions with only H_2_ in the gas phase. As for the other hydrogenotrophic metabolisms, their theoretical H_2_ consumption was estimated at between 20% and 65% when the gas phase was composed of the H_2_/CO_2_ mixture, and between 15% and 72% when only H_2_ was present. The taxonomic diversity results (Fig. 5) suggest that hydrogenotrophic sulfate reducers are the key players in this consumption. In deep aquifers with slow water turnover, low sulfate concentrations are expected to be rapidly consumed, allowing methanogens and acetogens to become the dominant metabolisms in a second phase. In the context of natural gas storage, annual monitoring at site Ab_L_1, the interface between stored natural gas and formation water, between 1992 and 2017 showed that increased microbial activity had reduced the sulfate concentration from 18 mg l^−1^ to less than 7 mg l^−1^ (190 to 73 µM; Ranchou-Peyruse et al. 2019). This increase in sulfate-reducing activity was explained by the solubilization of organic molecules present in the natural gas and available to heterotrophic microorganisms present in the water of the oligotrophic aquifer. This same research article also suggested that the effect of a massive arrival of H_2_ in such an ecosystem could impact microbial diversity, and by indirect effect on the physico-chemistry of water by maintaining low sulfate concentrations in particular, over several decades; and this even when the H_2_ storage was finished.

### Final microbial taxonomic diversity of cultivation trials with H_2_/CO_2_

Prokaryotic taxonomic diversity was studied at the end of incubation in biotic assays with a gas phase consisting of H_2_ or H_2_/CO_2_. These biomethanation conditions strongly selected for microbial communities, as previously reported (Bellini et al. 2022). In order to test a large number of conditions, it was decided not to use culture replicates for taxonomic diversity analyses, which can make it difficult, if not impossible, to interpret the evolution of complex microbial communities. Bearing this limitation in mind, we can only note the astonishing maintenance of a few prokaryotic genera present on all the sites tested, and that it is not possible to draw general conclusions on community behavior and changes without appropriate replication. The relative abundances of the 50 dominant ASVs obtained from high-throughput sequencing of the *16S rRNA* genes of the different communities are represented in the form of a heatmap (Fig. 5A). Although each condition tested was only in a single replicate for taxonomic diversity analyses, hydrogenotrophic methanogenesis was systematically carried out by members of the *Methanobacteriaceae* family, which includes the genera *Methanobacterium* and *Methanobrevibacter*, and the *Methanothermobacteriaceae* family (i.e. *Methanothermobacter* spp.), as confirmed by analysis of the *mcrA* genes in the same samples (Fig. S2, Supporting Information). The corresponding *16S rRNA* gene transcripts showed activity until the end of incubation (Fig. 5 B). These archaeal families are regularly highlighted in microbial communities in deep aquifers (Kotelnikova et al. 1998, Ma et al. 2019, Kadnikov at et al. 2020, Ranchou-Peyruse et al. 2019, 2021, Moliková et al. 2022) and were assumed to be responsible for *in situ* biomethanation in the case of town gas storage at the Lobodice site (Czechia; Buzek et al. 1994, Moliková et al. 2022). The growth conditions interfered with the representativeness of ASVs but ultimately had little influence on the results at the genus level. In the majority of trials, methanogenesis was carried out by members of the genus *Methanobacterium* (ASV16-16S, ASV4-16S, ASV13-16S, ASV25-16S, ASV46-16S, ASV48-16S, and ASV58-16S). In samples Ab_L_7, Ab_L_10, Ab_Y_2, and Ab_P_1, members of the *Methanothermobacter* genus were also represented. Their presence is unexpected, because of formation water temperatures at the bottom of the wells range from around 37°C to 40°C. These temperatures are deduced from temperature gradient measurements taken during logging operations (Gal et al. 2021). In 2019, archaea belonging to the *Methanopyraceae* family, a group of exclusively hyperthermophilic microorganisms, were identified at sites Ab_L_1, Ab_L_3, Ab_L_7, and Ab_L_10 (Ranchou-Peyruse et al. 2019). Faults allowing fluid circulation between the different superimposed aquifers could explain these results in the context of a sedimentary basin strongly impacted by the proximity of the Pyrenean mountain range and could explain the frequent detection of *a priori* strictly thermophilic organisms in the shallower mesothermal aquifers. However, this hypothesis does not explain why thermophilic microorganisms could be active and thrive at temperatures so far from these optima, even in this study with an incubation temperature of 37°C (Fig. 5). We hypothesize that these archaea are eurythermal or simply mesophile. The same was true of the order *Thermotogales*, which includes thermophiles. Environmental sequences of this order had been detected in mesothermal environments, such as a UGS in the Paris sedimentary basin (−830 m; Berlendis et al. 2010). Isolation from the aquifer’s formation water enabled to isolate a new species, *Mesotoga infera*, which can grow from 30°C to 50°C, with a growth optimum at 45°C (Ben Hania et al. 2013). Note that the *Methanobrevibacter* genus was also detected in formation water from well Ab_Y_2 (ASV49-mcr; Fig. S2, Supporting Information). Notably, the diversity study was carried out at the end of incubation when the sulfate had largely been consumed, i.e. under conditions that are *a priori* more favorable for methanogens than for sulfate reducers.

For the results based on the *16S rRNA* gene and its transcripts, while sulfate reducing conditions were constant in all cultivation trials, each enrichment culture seemed to be exclusively dominated by a phylogenetic group of sulfate reducers such as the genera *Desulfovibrio* (Ab_L_3, Ab_L_8, Ab_Y_2, and Ab_P_1), *Thermodesulfovibrio* (Ab_L_10) or *Desulforamulus* (Ab_L_1, Ab_L3, Ab_L_7, and Ab_L_10), suggesting competition between these different taxa (Fig. 5). The diversity of this group based on the *dsrB* gene (Fig. S3, Supporting Information) is more nuanced but could be explained by the persistence of spores in the assays and the greater specificity of the primers targeting the *dsrB* gene than the more generalist primers targeting the *16S rRNA* gene. This presumed higher specificity would also explain the detection of genera not identified by *16S rRNA*-based approaches (*Desulfosporosinus, Desulfosarcina, Desulfobulbus*, and LA-dsrAB), and therefore justifies the systematic use of the *dsrB* gene for the study of this functional group. Sporulating sulfate reducers are regularly found in deep continental environments and often described as lithoautotrophic (Aüllo et al. 2013). These bacteria are represented in all trials by one or two phylogenetic groups close to the genera *Desulfosporosinus, Desulfotomaculum*, and *Desulforamulus* or even the LA-dsrAB group (Müller et al. 2015). While these sulfate reducers have already been identified in this aquifer, some have also been identified in other UGS sites in aquifers, such as members of the *Desulforamulus* genus and microorganisms close to the strain formerly named *Desulfotomaculum profundi* Bs107 (Aüllo et al. 2016, Berlendis et al. 2016). In addition to these microorganisms, others persist in these simplified communities and have already been identified in a previous study carried out on this aquifer (*Burkhoderiaceae, Pseudomonadaceae*, and *Rhizobiaceae*; Ranchou-Peyruse et al. 2023). Their survival can be explained by a fermentative metabolism, as in the case of members of the genus *Pseudoclostridium* (ASV44-16S) and the phylogenetic group DTU014 (ASV68-16S; Dyksma et al. 2020). Under the conditions studied, no ASVs could be matched to any of the homoacetogenic bacteria previously described.

### Microbiological assessment of dedicated H_2_ storage in the lower Eocene

The formation water for well Ab_P_1 comes from a reservoir located at a lower level than the aquifer currently used as a UGS site, which itself evolved in a geological layer dating from the Eocene (Fig. 1 B). A rock sample from the same horizon as Ab_P_1, at the boundary of the Eocene and Dano-Paleocene, was obtained and used in the tests in place of calcite. This rock is composed of 63% quartz, 13% calcite, 16% clay, and 7% pyrite (DRX/FluoX analysis, TEREGA data). During the first sampling campaign, this formation water had one of the highest concentrations of methanogenic archaea, with 2.62 × 10^2^ ± 5.7 × 10^1^  *mcrA* gene copies ml^−1^. The methane production from Ab_P_1 formation water in the presence of H_2_/CO_2_ gas (80/20; 1 bar) was among the highest and did not increase in the presence of rock (Fig. 4; Fig. S4, Supporting Information). After 2 months of rock-free incubation with a gas phase composed of H_2_/CO_2_, the test carried out with formation water from site Ab_P_1 showed a production of 3.3 × 10^−1^ mmol of CH_4_ in 2 months with a total consumption of 3.8 mmol of H_2_. With rock incubation, H_2_ consumption almost doubled (6.4 mmol) and CH_4_ production decreased (1.4 × 10^−1^ mmol), revealing increased activity of metabolisms other than methanogenesis. Conversely, when the gas phase was composed solely of H_2_ (1 bar), the yield was among the lowest. For the other sites, the highest pH values were obtained in the absence of CO_2_ in the gas phase and were associated with the lowest Ca^2+^ and HCO_3_^−^ concentrations (Fig. S4 and Table S2, Supporting Information).

The results presented in Fig. S5 (Supporting Information) clearly illustrate the strong similarity between the taxonomic diversity obtained from the *16S rRNA* genes and that obtained from their transcripts. For batch cultures with very limited available nutrients, this result is interesting, as it suggests a restructuring of the microbial community with strong recycling of the necromass constituted by microorganisms that are not adapted to the experimental conditions and leave no remnant DNA. From an initial state mainly dominated by sporulating sulfate-reducing Firmicutes affiliated with the *Desulfurispora* genus, the communities were subsequently all dominated by hydrogenotrophic methanogenic archaea belonging to the *Methanobacteriaceae* or *Methanothermobacteriaceae* families. The results suggest that the members of *Methanothermobacteriaceae* are not all thermophilic since the environmental factor selecting them was not temperature, but rather the acid pH induced by the addition of CO_2_ associated with one or more nutrients released into the rock.

We note that while the addition or nonaddition of calcite or rock did not have any effect on the structuring of microbial communities based on H_2_ and CO_2_ consumption and production and dominated by methanogens and sulfate reducers. Regarding calcite, rock, or even barite supplementation, the diversity may differ between communities at the ASV level, but this variation is very low, or even nonexistent, at the microbial genus level. However, these minerals represent a carbon source (calcite dissolution), sulfate source (barite dissolution), and buffer for microorganisms, they had little impact on the structure of the sulfate-reducing functional group and none on that of methanogens. These results suggest that the ecological valence of these microorganisms is stronger than expected. For example, members of the genus *Methanobacterium* show activity at pH values ranging from ∼6 (conditions with H_2_/CO_2_) to around pH 10. While alkalinization is often associated with methanogenesis and sulfate reduction, a sharp increase in pH has been shown to be responsible for the cessation of methanogenesis. Here, methane production yields were lower when the gas phase was composed solely of H_2_ (without CO_2_), even in the presence of calcite as an indirect carbon source. On deep aquifers with mineralogically more complex reservoir rocks, a recent study experimentally simulating H_2_ injections into a high-pressure three-phase reactor (gas–rock–water) with indigenous microorganisms suggested similar alkalinization during physicochemical modeling (Mura et al. 2024). Here, the rock of the aquifer studied is essentially composed of quartz (81%), while calcite was estimated at around 12% (Haddad et al. 2023). It is reasonable to assume that the buffering effect of the aquifer rock is greater than that of our test media, but over the lifetime of such a storage facility (i.e. several decades), it seems likely that minerals such as calcite will be almost completely dissolved, given their low concentrations. On the other hand, bearing in mind that even at the highest pH and based on the study of the *16S rRNA, dsrB*, and *mcrA* genes transcripts, methanogenic archaea continued to be active, we hypothesize that the low methane yields may be more related to a limitation of CO_2_ solubilization rather than to a deleterious effect of pH on the physiological activity of the hydrogenotrophs present.

## Conclusion

As the first study of its kind on this aquifer, which serves as a UGS for natural gas, these experiments are intended to assess the hydrogenotrophic potential of indigenous communities in general, and of methanogens in particular. Interestingly, it was shown that the hydrogenotrophy capacity linked to sulfate reduction was present over the entire aquifer used as UGS and hydrogenotrophic methanogenesis was only present near the current natural gas storage. It is obvious than these batch experiments at atmospheric pressure underestimate H_2_ consumption because of its low dissolution and low quantity available for the microbial growth. However, this study has made it possible to identify certain sites and conditions to be tested from now on under conditions closer to reality (high pressure, monitoring over time, and so on) in order to determine H_2_ consumption (or even CO_2_) and methane and sulfide production yields, and to assess the economic relevance of a future UHS in this deep aquifer. Finally, the strong alkalization initiated by lithoautotrophic microbial metabolisms is a key parameter to take into consideration. In the context of a UHS sure, this phenomenon could considerably curb microbial consumption of H_2_ by mineralizing CO_2_ dissolved in carbonates and thus making this CO_2_ inaccessible to autotrophic microorganisms. In the context of *in situ* biomethanation, alkalinization could be counterbalanced by CO_2_ coinjection, enabling active *in situ* biomethanation to be maintained.

## Supplementary Material

fiae066_Supplemental_Files
