# Supplementary material for: Assessment of the in situ biomethanation potential of a deep aquifer used for natural gas storage
Source: FEMS Microbiol Ecol. 2024 Apr 24;100(6):fiae066. doi: 10.1093/femsec/fiae066 (PMC11092278; doi:10.1093/femsec/fiae066)
Supplement: fiae066_Supplemental_Files [file fiae066_supplemental_files.zip › Table supp data_S2_final (1).docx]

**Table S2: Physico-chemical data used in Figures 3 and S3.**
