# Supplementary material for: Assessment of the in situ biomethanation potential of a deep aquifer used for natural gas storage
Source: FEMS Microbiol Ecol. 2024 Apr 24;100(6):fiae066. doi: 10.1093/femsec/fiae066 (PMC11092278; doi:10.1093/femsec/fiae066)
Supplement: fiae066_Supplemental_Files [file fiae066_supplemental_files.zip › Table supp data_S3_final (1).docx]

**Table S3: Comparison of prokaryote (bacteria and archaea) quantifications in the seven formation waters selected in the second step of the study with different conditions of incubation.** Concentrations of prokaryotes, sulfate reducers and methanogens were estimated by qPCR in copy numbers per milliliter of water of the *16S rRNA*, *dsrB* and *mcrA* genes and transcripts, respectively.

N/A : no more nucleic acids
