# Supplementary figures and images for: Assessment of the in situ biomethanation potential of a deep aquifer used for natural gas storage
Source: FEMS Microbiol Ecol. 2024 Apr 24;100(6):fiae066. doi: 10.1093/femsec/fiae066 (PMC11092278; doi:10.1093/femsec/fiae066)

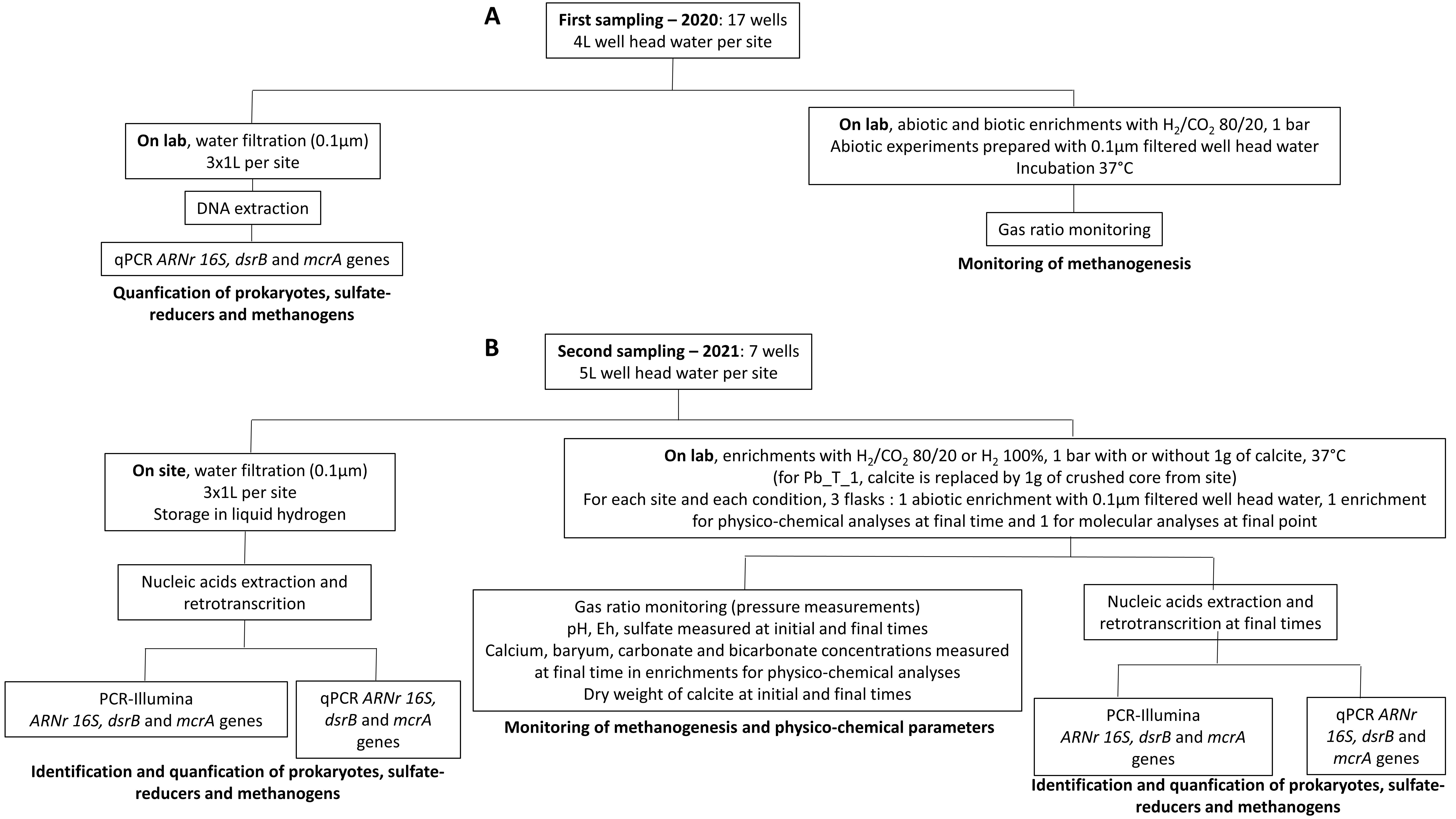

Supplement: fiae066_Supplemental_Files [file fiae066_supplemental_files.zip › Figure supp data S1_17_04_24 (1).jpg]

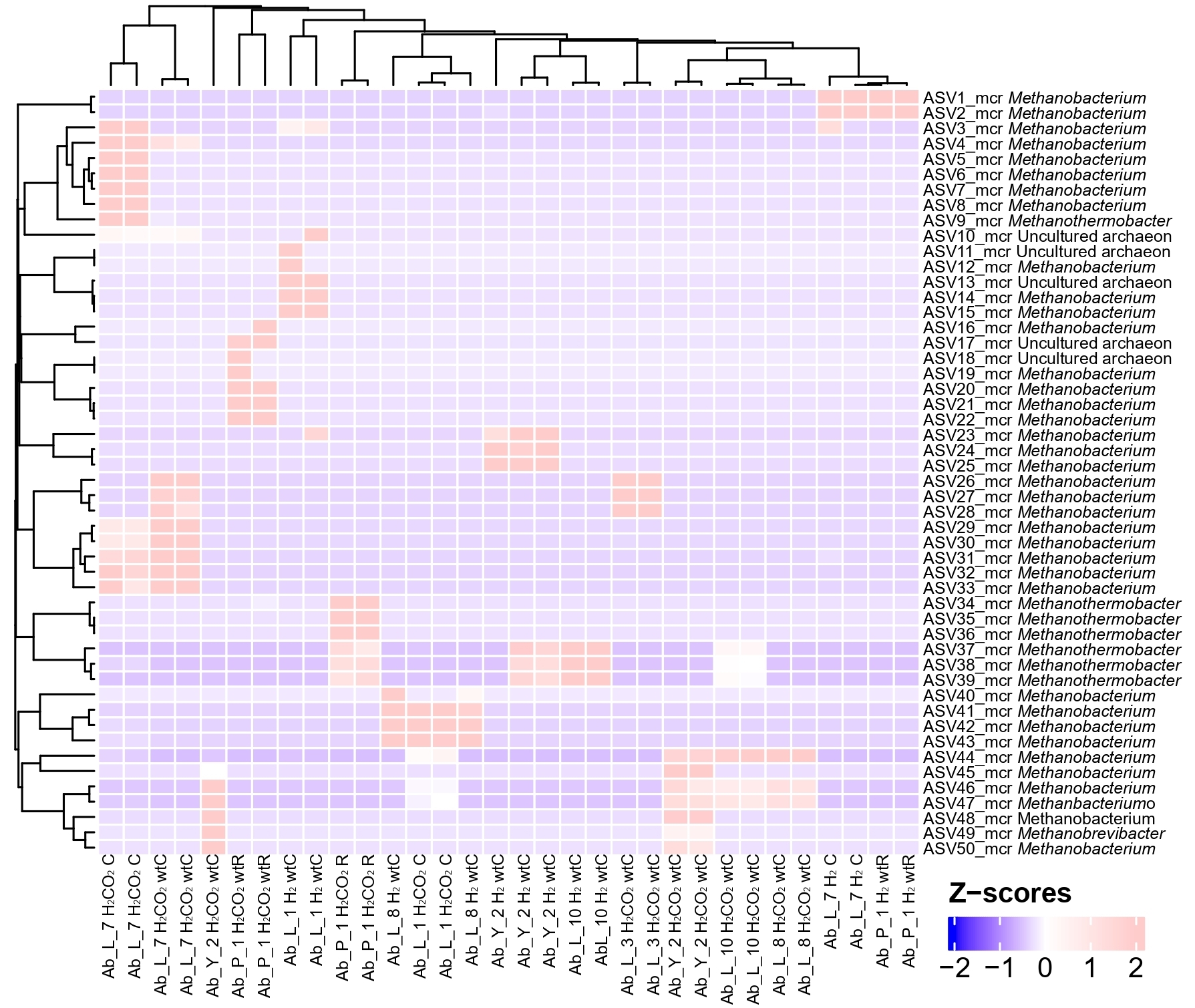

Supplement: fiae066_Supplemental_Files [file fiae066_supplemental_files.zip › Figure supp data_S2__final (1).jpg]

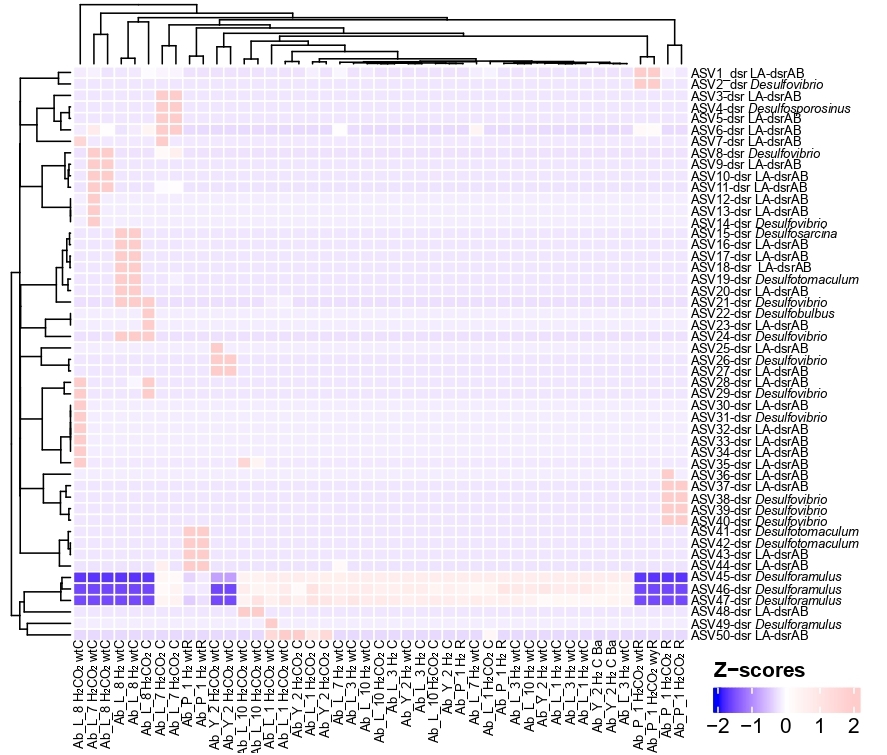

Supplement: fiae066_Supplemental_Files [file fiae066_supplemental_files.zip › Figure supp data_S3_final (1).jpg]

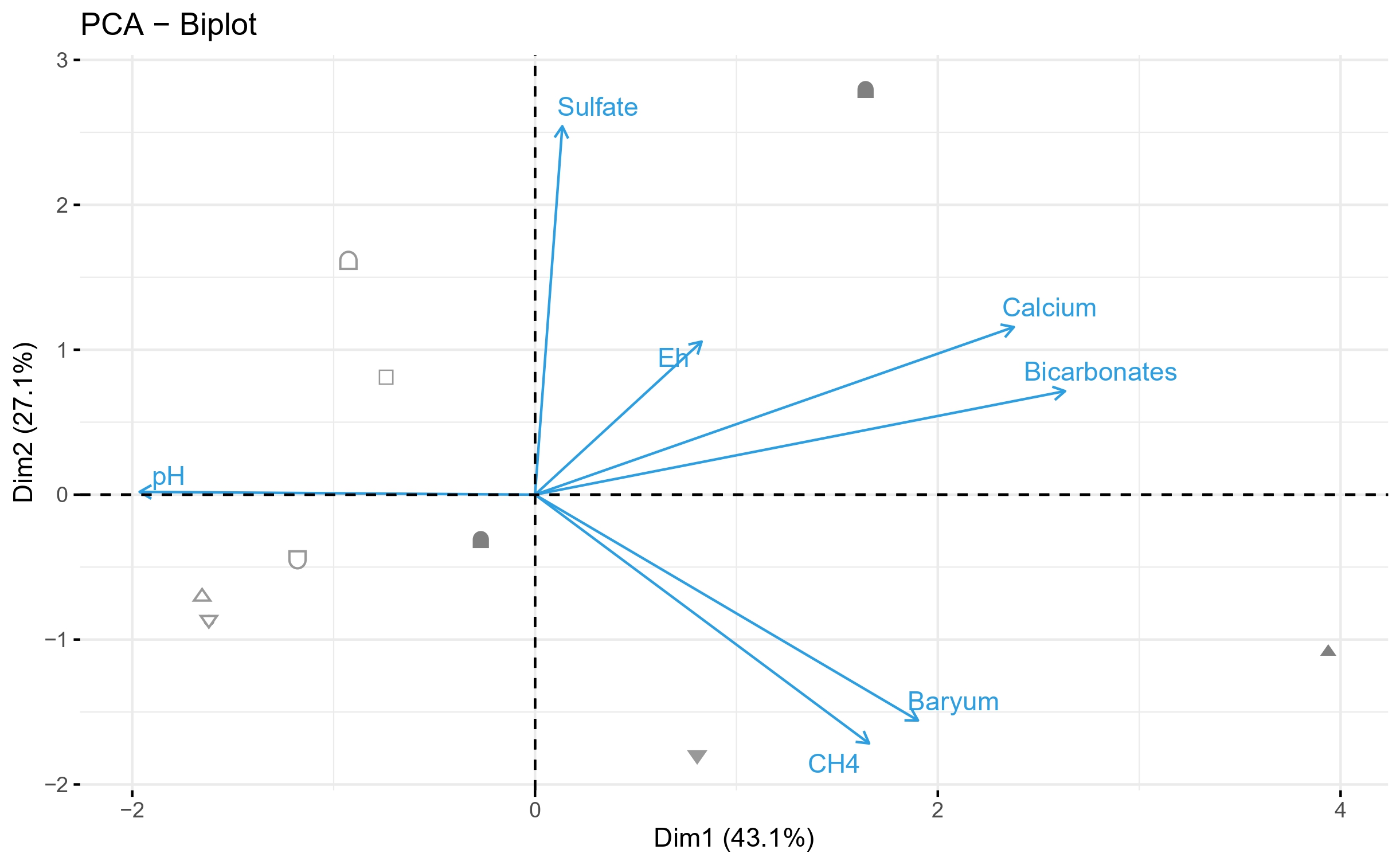

Supplement: fiae066_Supplemental_Files [file fiae066_supplemental_files.zip › Figure supp data_S4_Final (1).jpg]

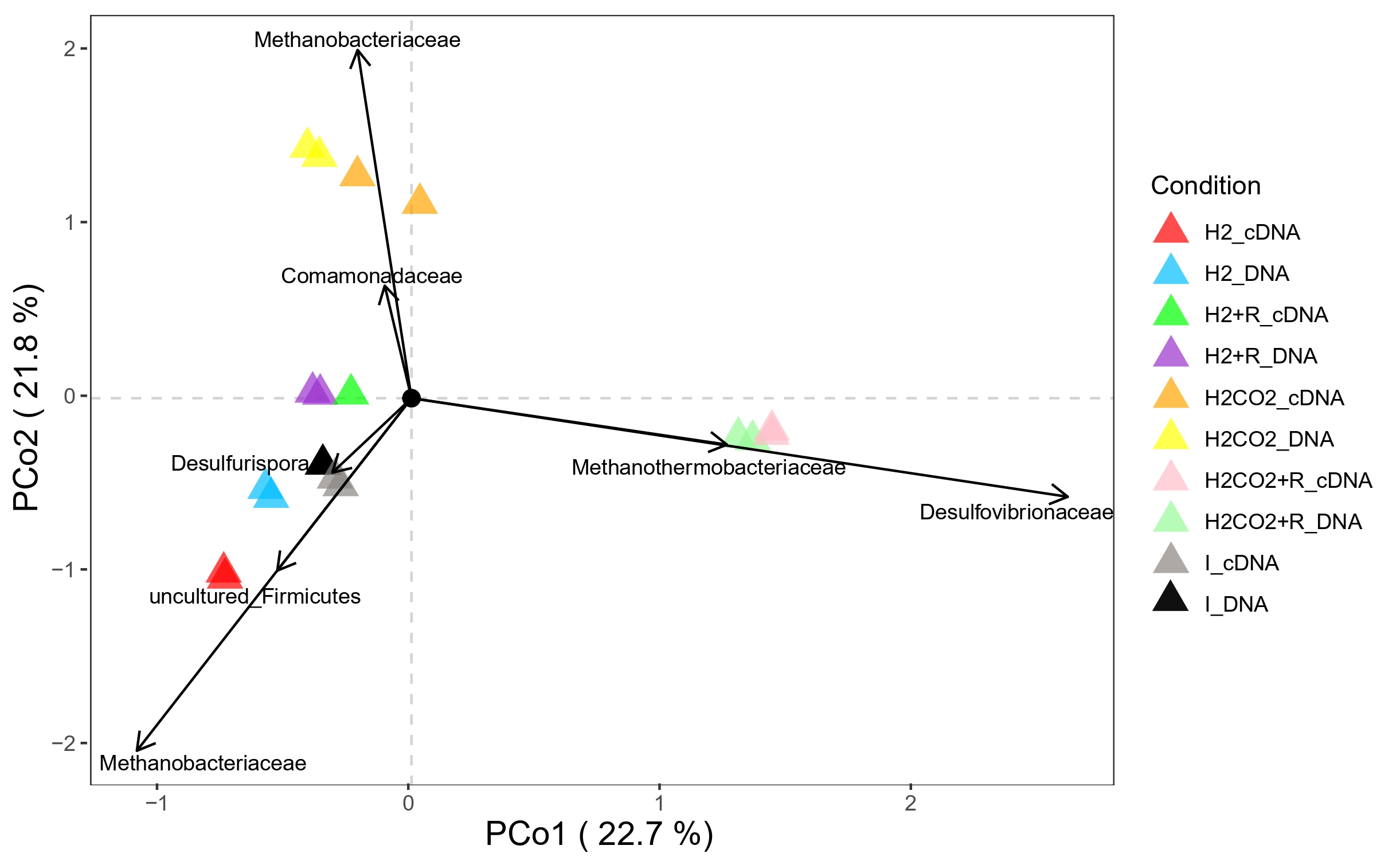

Supplement: fiae066_Supplemental_Files [file fiae066_supplemental_files.zip › Figure supp data_S5_final (1).jpg]
